# Non-medical financial burden in tuberculosis care: a cross-sectional survey in rural China

**DOI:** 10.1186/s40249-016-0101-5

**Published:** 2016-01-26

**Authors:** Qiang Li, Weixi Jiang, Quanli Wang, Yuan Shen, Jingyuan Gao, Kaori D. Sato, Qian Long, Henry Lucas

**Affiliations:** Department of Biostatistics and Epidemiology, School of Public Health, Xi’an Jiaotong University, Xi’an, Shaanxi China; Global Health Research Center, Duke Kunshan University, Kunshan, Jiangsu China; Duke Global Health Institute, Duke University, Durham, NC USA; Institute of Development Studies at the University of Sussex, Brighton, UK

**Keywords:** Non-medical cost, Financial burden, Tuberculosis, China

## Abstract

**Background:**

Treatment of tuberculosis (TB) in China is partially covered by national programs and health insurance schemes, though TB patients often face considerable medical expenditures. For some, especially those from poorer households, non-medical costs, such as transport, accommodation, and nutritional supplementation may be a substantial additional burden. In this article we aim to evaluate these non-medical costs induced by seeking TB care using data from a large scale cross-sectional survey.

**Methods:**

A total of 797 TB cases from three cities were randomly selected using a stratified cluster sampling design. Inpatient medical costs, outpatient medical costs, and direct non-medical costs related to TB treatment were collected using in-person interviews by trained interviewers. Mean and median non-medical costs for different sub-groups were calculated and compared using Kruskal-Wallis and Mann–Whitney U tests. Regression analysis was conducted to assess the influence of different patient characteristics on total non-medical cost.

**Results:**

The median non-medical cost was RMB 1429, with interquartile range RMB 424–2793. The median non-medical costs relating to inpatient treatment, outpatient treatment, and additional nutrition supplementation were RMB 540, 91, and 900, respectively. Of the 797 cases, 20 % reported catastrophic expenditure on non-medical costs. Statistically significant differences were detected between different cities, age groups, geographical locations, inpatient/outpatient care, education levels and family income groups.

**Conclusions:**

Non-medical costs relating to TB treatment are a serious financial burden for many TB patients. Financial assistance that can limit this burden is urgently needed during the treatment period, especially for the poor.

**Electronic supplementary material:**

The online version of this article (doi:10.1186/s40249-016-0101-5) contains supplementary material, which is available to authorized users.

## Multilingual abstracts

Please see Additional file 1 for translations of the abstract into the six official working languages of the United Nations.

## Background

China has the second largest burden of tuberculosis (TB) in the world, accounting for 12 % of all cases [1]. Although China more than halved its TB prevalence rate from 1990 to 2010 [2], the rate remained high at the end of this period, at 459 per 100,000 for a population over 15 years old, implying significant social and economic burdens [3]. Prevalence rates are higher in poor, rural areas [4] and the poor have less access to TB care and are less likely to be cured [5]. Substandard living conditions, underlying health problems, malnourishment, a lack of money to pay for health care and inadequate access to health services all play major roles in impeding the successful treatment of TB [6].

The cost of inpatient treatment of TB in China is partly funded by health insurance schemes for those enrolled, and outpatient treatment is funded by China’s national TB control program [7]. Although out of pocket (OOP) payments may also be partially reimbursed by local programs in some areas, patients must meet the largest share of outpatient care expenditures [8]. A number of studies have found that the financial burden relating to treatment was the most cited reason for default [9], and that non-medical costs constituted a substantial portion of this burden [10]. Treatment typically lasts six months and patients make six trips to their outpatient clinics, potentially incurring travel and accommodation costs during treatment. While this is the standard number of visits, a full course of treatment may be delivered with a minimum of four visits where patients live a considerable distance from the facility. These non-medical costs typically include payments for transport, accommodation, and the cost of nutritional supplementation during the treatment period.

Previous international studies have examined the financial burden of non-medical costs and their impact on adherence to treatment. One systematic review of overall costs for TB patients has shown that non-medical cost accounted on average for 20 % of total expenditure [11]. Other studies have found that some TB patients may be discouraged from seeking care or adhering to treatment plans by non-medical costs [12, 13]. Transport and accommodation costs are most often considered, but one study of hospitalization for TB in Ghana, Vietnam, and the Dominican Republic indicates the substantial burden of additional food costs during treatment [14]. In China, while numerous studies have investigated the financial burden on TB patients [15–18], some others have focused on non-medical costs or the factors that influence them [16, 19], such as residence location, gender, age, inpatient versus outpatient care, health insurance status, education level, family income, and patient category.

Here, a large-scale cross-sectional survey in three Chinese cities was used to assess the non-medical financial burden on TB patients relating to expenditures on transportation, accommodation and supplementary nutrition. We also analyzed the factors influencing these expenditures.

## Methods

We designed and conducted a cross-sectional survey in TB patients. In China, the administrative demarcations move downward from country to provinces to prefectures/cities to districts/counties and to towns. The study was undertaken in Zhenjiang City, Jiangsu Province in eastern China; Yichang City, Hubei Province in central China; and Hanzhong City, Shaanxi Province in western China. Sample size calculations indicated that a minimum of 792 TB cases (264 in each city) were necessary as the assumed sample proportion of catastrophic expenditure on non-medical costs were 20 %, with 5 % as half the width of the confidence interval, and α = 0.05. In each city one county/district was randomly selected by a random number from each category of those with high, middle and low GDP per capita. Three townships/streets were then selected at random in each selected county/district and thirty TB cases were randomly selected from each township/street using a list of registered cases. Patients who completed normal treatment or stopped treatment during 2012 were included in the study. We excluded patients with communication barriers, such as those with hearing impairments. We also excluded patients with serious diseases and migrant workers who did not join the survey within the study period. Patients with mental illnesses were excluded as well. In total, 797 TB patients were recruited and informed consent of each participant was obtained.

Interviews were conducted by trained enumerators using a structured questionnaire to collect the medical and non-medical costs (transportation, accommodation, and nutritional supplementation) of TB treatment. Information regarding personal demographic and socio-economic status (age, sex, education, family income/expenditure, etc.; Table 1), reimbursements from health insurance, and financial assistance from government agencies was also collected. The field survey was conducted between April 2013 and May 2013.

**Table 1:** Basic characteristics of TB patients according to study site ^a^

Characteristic		Hanzhong	Yichang	Zhenjiang	Total
Age in year, (Mean ± SD^b^, years)		55.8 ± 14.4	53.6 ± 15.0	59.3 ±14.7	56.2 ±14.9
Sex	Male	211 (78.1)	191 (72.3)	192 (73.0)	594 (74.5)
	Female	59 (21.9)	73 (27.7)	71 (27.0)	203 (25.5)
Family size, (Mean ± SD, people)		3.1 ± 1.4	3.0 ± 1.4	3.4 ±1.7	3.2 ±1.5
Area	Rural	252 (93.3)	249 (94.3)	235 (89.4)	736 (92.3)
	Urban	18 (6.7)	15 (5.7)	28 (10.6)	61 (7.7)
Patient category	New patient	218 (80.7)	227 (86.0)	193 (73.4)	638 (80.1)
	Relapse patient	43 (15.9)	37 (14.0)	68 (25.9)	148 (18.6)
	Failure of previously untreated patient	5 (1.9)	0 (0.0)	2 (0.8)	7 (0.9)
	Failure of re-treated patient /chronic	4 (1.5)	0 (0.0)	0 (0.0)	4 (0.5)
Education	Never attended school	75 (27.8)	30 (11.4)	58 (22.1)	163 (20.5)
	Primary school	85 (31.5)	91 (34.5)	86 (32.7)	262 (32.9)
	Junior high school or at the same level	89 (33.0)	96 (36.4)	84 (31.9)	269 (33.8)
	High school or at the same level	21 (7.8)	47 (17.8)	35 (13.3)	103 (12.9)
Health insurance	With health insurance	267 (98.9)	260 (98.5)	257 (97.7)	784 (98.4)
	Without health insurance	3 (1.1)	4 (1.5)	6 (2.3)	13 (1.6)
Family income, (Median (P25–P75^b^), RMB 1000^c^)		15.0 (5.0–30.0)	20.0 (9.2–32.6)	36.0 (13.0–63.6)	21.4 (7.6–40.0)
Family expenditure, (Median (P25–P75^b^), RMB 1000^c^)		15.0 (7.5–23.5)	20.0 (9.0–30.0)	20.0 (10.0–35.0)	20.0 (9.8–30.0)

^a^Data are n(%) unless otherwise indicated. TB: tuberculosis

^b^SD: standard deviation. P25: 25^th^ percentile. P75: 75^th^ percentile

^c^RMB 1000: a currency exchange rate of Chinese RMB 628 Yuan to US$100 Yuan at the end of 2012,RMB1000 = US$159

Only patients with a ‘confirmed’ TB diagnoses were included. Most had at least one sputum smear test and one chest x-ray. Indirect expenditures on transport and accommodation incurred by patients, their families and others related to seeking and accessing TB treatment during pre-diagnostic, diagnostic and post-diagnostic periods, as well as during hospitalization where applicable, were recalled by patients and their caregivers,. The cost for nutritional supplementation during TB treatment was estimated by extracting the cost of extra food expenditure (such as meat, milk, vitamins, etc.). We attempted to minimize the recall bias via in-depth interviews with the patient.

Ethical approval was sought and granted for this research by the Ethical Committee of China CDC. It was recognized that the right and the welfare of the subject were adequately protected; the potential risks were outweighed by the potential benefits. The ethical approval number was 201307.

### Statistical analysis

We quantified non-medical costs by aggregating the transport, accommodation, and nutritional supplementation expenditures related to TB health care. Overall, 752 patients reported complete non-medical costs for transport, accommodation, and nutritional supplementation, while others missed some portion of the above. Cases with missing data were deleted when analyzing the corresponding costs. Mean and median non-medical expenditures were calculated and compared across subgroups using Mann–Whitney U and Kruskal-Wallis tests and a 5 % significance level. Linear regression was then used to model the relationships between non-medical costs and the explanatory variables available from the survey data. We also separated the transport plus accommodation costs and the additional nutrition cost for the multi-variate analysis. All the statistical analysis was done using the SAS version 9.3 statistical software package (SAS Institute Inc., Cary, North Carolina).

We considered the following patient variables to be potentially correlated with non-medical costs as they were major risk factors for the non-medical cost: residence location (the three study cities), gender, age (<65 years or > =65 years), residence type (urban or rural), inpatient care (with or without), health insurance (covered or uncovered), education level (never attended school, primary school, junior high school, high school or higher), family income (as a proportion of the median in each city), and patient category (new or relapse patient).

## Results

Table 1 shows the basic demographic characteristics of the participants. In total, 797 TB patients were included in the study, with mean ages ranging from 53.6 to 59.3 across the three study sites. Some 74.5 % were male, and 80.1 % were new patients. Most came from rural areas, and their degree of education was limited. Overall one-fifth (almost 28 % in Hanzhong) had no formal education. Only 12.9 % of the participants had received a high school education or similar. The average family income was RMB 21,400 (equaling to US$3408, a currency exchange rate of Chinese RMB 628 Yuan to US$100 at the end of 2012), ranging from RMB 15,000 (US$2389) in Hanzhong to RMB 36,000 (US$5732) in Zhenjiang. It was notable that the average family expenditure (RMB 20,000) was almost equal to the average income, indicating that many families would have very limited ability to save. As expected, given the government promotion of the urban and rural health insurance schemes, almost all patients were covered by one of these schemes. The largest percentage (41.9 %) of participants made six trips to their outpatient clinic during treatment, potentially incurring travel and accommodation costs.

As shown in Table 2, non-medical financial costs related to TB treatment varied considerably across study sites. The overall mean and median expenditure was considerably lower in Yichang than in Hanzhong and Zhenjiang. However, this was a simply reflection of the much more limited expenditure on supplementary nutrition – around half that in the other cities. Both travel and accommodation costs were substantially higher in Yichang, which was probably a reflection of the increased distance that patients had to travel to a designated TB facility.

**Table 2:** Non-medical financial cost related to TB treatment by study site (RMB^a^)

	n	Hanzhong			Yichang			Zhenjiang			Total		
		Mean	Median	P25-P75^b^	Mean	Median	P25-P75	Mean	Median	P25-P75	Mean	Median	P25-P75
Total	752^c^	2308	1785	853-3185	1453	968	318-1880	2061	1800	381-3000	1943	1429	424-2793
Inpatient	371												
Travel fee		130	30	10-100	257	50	20-270	124	15	0-100	158	30	6-120
Accommodation fee		822	530	150-1060	1018	760	300-1350	547	315	108-630	766	500	150-1000
Subtotal		958	600	210-1240	1285	815	350-1710	673	400	200-823	928	540	208-1200
Outpatient	753												
Travel fee		98	60	24-120	150	120	40-204	77	28	0-96	109	60	12-140
Accommodation fee		124	30	0-100	56	20	0-75	20	0	0-0	68	0	0-60
Subtotal		223	91	30-240	206	144	60-270	97	36	0-120	176	91	20-210
Additional nutrition fee	770	1586	1200	300-2400	828	435	0-1200	1596	1200	0-2400	1337	900	0-1800

^a^RMB 1000: a currency exchange rate of Chinese RMB 628 Yuan to US$100 Yuan at the end of 2012, RMB1000 = US$159

^b^P25: 25^th^ percentile. P75: 75^th^ percentile

^c^The total non-medical cost is the sum of the inpatient cost, outpatient cost and the additional nutrition fee. The total number of patients is less than that in the subgroup because of missing data

Table 3 reveals the non-medical economic burden of TB care, as measured by the numbers/proportions of patients with catastrophic health care expenditure on care. Overall, some 20 % of all respondents reported that their non-medical costs exceeded 40 % of their non-food expenditure, while 37 % reported that these costs exceeded 10 % of their annual household income. The non-medical burden was highest in Hanzhong using both measures.

**Table 3:** Description of the non-medical burden of TB health care, n (%)

Non-medical cost in TB health care	Hanzhong	Yichang	Zhenjiang	Total	*P*_value ^a^
≥ 40 % of the annual capacity to pay	65 (26.9)	29 (11.9)	51 (21.2)	145 (20.0)	<0.011
≥ 10 % of the annual household income	132 (52.4)	81 (32.7)	62 (25.3)	275 (36.9)	<0.011
Groups of non-medical cost (RMB)					
≤ 200	35 (13.8)	45 (18.1)	53 (21.3)	133 (17.7)	<0.011
201 ~ 1000	45 (17.7)	82 (32.9)	31 (12.4)	158 (21.0)	
1001 ~ 2000	64 (25.2)	64 (25.7)	60 (24.1)	188 (25.0)	
2001 ~ 4000	66 (26.0)	38 (15.3)	70 (28.1)	174 (23.1)	
4001 ~ 6000	29 (11.4)	14 (5.6)	26 (10.4)	69 (9.2)	
≥ 6001	15 (5.9)	6 (2.4)	9 (3.6)	30 (4.0)	

^a^ Chi-square test was used to compare the difference of percentage between study sites

Table 4 shows the influence of different patient characteristics on total non-medical costs. In addition to the variation between the study cities, the relationships between non-medical costs and age group, residence type (urban/rural), receiving inpatient care, education and family income were statistically significant.

**Table 4:** Comparison of non-medical cost of TB care between different characteristics, RMB

Characteristic	Mean	Median	*P*_value ^a^
City			<0.001
Hanzhong	2308	1785	
Yichang	1453	968	
Zhenjiang	2060	1800	
Sex			0.445
Men	1940	1410	
Women	1950	1470	
Age			<0.001
< 65 years	2163	1605	
> =65 years	1490	1050	
Residence type			<0.001
Urban	3128	2600	
Rural	1842	1320	
Inpatient care			<0.001
With	2567	2115	
Without	1370	900	
Health insurance			0.889
Covered	1794	1495	
Uncovered	1945	1429	
Education			<0.001
Never attended school	1389	1040	
Primary school	1864	1268	
Junior high school	2146	1658	
High school or higher	2479	1837	
Family income			<0.001
Lower half	1560	1066	
Higher half	2298	1700	
Category			0.540
New patient	1884	1406	
Relapse patient	2182	1600	

^a^ *P*_value was calculated by univariate analysis

The results of the regression analysis are reported in Table 5. In this analysis we combined transportation and accommodation costs as both are related to geographic factors such as distance from home to facility and availability of transportation options. The table indicates that after controlling for other variables, living in Yichang was still associated with higher transportation and accommodation costs and lower additional nutrition costs. For transportation and accommodation costs specifically, only care type and age were likely to have a significant influence in addition to city of residence. However, additional nutrition costs were also positively correlated with higher education level, family income and urban residence.

**Table 5:** Regression analysis of total non-medical cost and patient characteristics ^a^

Variable		Total non-medical cost			Transportation plus accommodation cost			Additional nutrition cost		
		Parameter	SE	*P* value	Parameter	SE	*P* value	Parameter	SE	*P* value
Location										
Hanzhong		8.00	1.86	<.0001	−1.98	1.19	0.0970	13.31	2.02	<.0001
Yichang	Ref									
Zhenjiang		4.45	1.86	0.0167	−6.42	1.19	<.0001	10.87	2.02	<.0001
Sex	Ref: male	2.66	1.73	0.1241	0.40	1.11	0.7203	2.31	1.89	0.2209
age	Ref: aged <65	−6.12	1.70	0.0003	−2.38	1.08	0.0285	−5.24	1.84	0.0046
Residence type	Ref: rural	10.18	2.82	0.0003	−0.62	1.83	0.7356	11.35	3.05	0.0002
Inpatient care	Ref: without	14.86	1.50	<.0001	18.53	0.96	<.0001	4.00	1.62	0.0140
Health insurance	Ref: covered	7.12	5.91	0.2290	6.88	3.84	0.0737	1.86	6.22	0.7657
Education										
Never attended school	Ref									
Primary school		6.22	2.15	0.0040	0.31	1.37	0.8197	6.47	2.33	0.0057
Junior high school		7.32	2.24	0.0011	1.36	1.43	0.3432	6.49	2.44	0.0079
High school or higher		9.19	2.90	0.0016	0.27	1.84	0.8815	9.25	3.15	0.0034
Family income	Ref: lower half	4.12	1.57	0.0090	0.45	1.00	0.6517	6.16	1.70	0.0003
Category	Ref: new patients	1.61	1.87	0.3891	−1.54	1.19	0.1983	2.69	2.02	0.1838

^a^ Following variables were enrolled in the regression medel: residence location (the three study cities), gender, age (<65 years or > =65 years), residence type (urban or rural), inpatient care (with or without), health insurance (covered or uncovered), education level (never attended school, primary school, Junior high school, high school or higher), family income (as a proportion of the median in each city), and patient category (new or relapse patient)

## Discussion

China’s national TB control program requires patients to visit the TB outpatient clinic every month for six months or at least four times during the first, second, fifth and last month of the treatment regimen [20]. A majority of participants in this study adhered to this requirement, although many of them suffered from heavy financial burden caused by TB treatment. In many cases, especially where patients lived in more remote areas – which were typically associated with lower household incomes – this entailed considerable expenditure on travel and accommodation, leading to non-medical expenditures that may be comparable to out-of-pocket payments for hospitalization.

Non-medical costs of TB treatment placed a considerable financial burden on patients. Some 25 % of participants spent RMB 1001 ~ 2000 on non-medical costs while over 23 % spent RMB 2001 ~ 4000. These costs were considerable when compared to household expenditures, which ranged from RMB 15,000 to 20,000 across the three cities, and to incomes, which ranged from RMB 15,000 to 36,000.

Inpatient care was positively associated with both types of costs (*P* < 0.05), which can be explained by the fact that receiving inpatient care increased the accommodation costs for patients and their companions, and the likelihood of purchasing additional food during hospitalization. The negative impacts of higher age on both cost components may be ascribed to older people’s limited ability to travel to health facilities (especially for patients in remote areas) and to their lower willingness and capacity to pay for additional food. This is consistent with results from previous studies which revealed that older people do not want use their children’s money for treatment as they believe the money could be used for more meaningful purposes, for example, the education of their grandchildren [16, 21].

The impact of other patient characteristics on non-medical costs were mixed for the two types of costs. Geographic constraints played a major role in the influence of location on costs, as Yichang and Hanzhong have vast mountainous areas, which increases travel costs and adds to the difficulty of purchasing additional food. Indicators of socio-economic status – residence type, education and family income – were all positively associated with additional nutrition costs but had no significant influence on travel and accommodation costs. This would suggest that patients with higher levels of education, a greater ability to pay and wider availability of nutritional supplements (in urban areas) were more willing and able to consume more nutritious foods to help with recovery.

The results of the regression analyses revealed the different nature of the two types of non-medical costs, and suggested how to reduce patient costs in following the prescribed treatment regimen. Future policies might best serve to focus on reducing travel and accommodation costs, which have the greatest impact on poorer households in remote areas. One study has confirmed the role of transportation subsidies in reducing the financial burden of TB patients but suggests that the amount provided needs to be more substantial [19, 22]. Our study indicates that geographic factors exert such a major influence on non-medical costs that the amount of travel subsidy should be determined by location of residence. Additional subsidies may also be appropriate to encourage adherence to treatment by older patients. For the effect of health insurance, our results were similar to a previous study which indicated higher non-medical costs for those not covered by any health insurance [16].

This study is not without its limitations. Firstly, investigators were trained to extract information as reliably as possible. However, the recall bias of annual income and non-direct medical costs can hardly be avoided due to the long treatment duration. Patients’ estimation of transportation, accommodation and additional food costs may not be accurate. Secondly, some patients received therapy mainly in 2011 and so major non-medical costs were incurred in 2011; however, these patients reported their 2012 income which may not have reflected their economic statuses during treatment when incomes may have been reduced. In addition, only three cities form eastern China, central China and western China were selected by location instead of by TB burden level. Cities with the highest TB burden, such as Tibet, Xinjiang and Guizhou, were not involved. Thus, the present results were limited when considering TB burden levels. Future studies on the economic burden of non-medical costs could gather more accurate information through timely monitoring during treatment in broader areas.

## Conclusion

Non-medical costs related to TB treatment imposed a considerable financial burden for TB patients, often accounting for a considerable proportion of their annual income. Geographic factors played such an important role in transportation and accommodation costs that transportation subsidies should be provided based on the patients’ places of residence.

## Additional file

Additional file 1:
**Multilingual abstracts in the six official working languages of the United Nations.** (PDF 542 kb)

## Abbreviations

GDP: Gross Domestic Product
OOP: Out-of-Pocket payment
TB: Tuberculosis
