# Supplementary material for: Non-medical financial burden in tuberculosis care: a cross-sectional survey in rural China
Source: Infect Dis Poverty. 2016 Jan 26;5:5. doi: 10.1186/s40249-016-0101-5 (PMC4727322; doi:10.1186/s40249-016-0101-5)
Supplement: Additional file 1: — Multilingual abstracts in the six official working languages of the United Nations. (PDF 542 kb) [file 40249_2016_101_MOESM1_ESM.pdf]

Translation of the abstract into the six official working languages of the United Nations

الأعباء المادية غير الطبية في مجال رعاية مرضى السل: دراسة مقطعية في المناطق الريفية في الصين

كيانغ لي، ويكسي جيانغ، كوانلي وانغ، يوان شين، جينغيوان جاو، كاوري د. ساتو، كيان لونغ، هينري لوكاس

ملخص

معلومات تمهيدية: على الرغم من أن نفقات علاج السل في الصين تُغطّى جزئياً من قبل البرامج الوطنية وأنظمة التأمين الصحي، فإن مرضى السل يتحمّلون أعباءً طبية مرتفعة. فبالنسبة للبعض منهم، وخاصة أولئك الذين ينتمون إلى طبقات فقيرة، تشكل النفقات غير الطبية، مثل التنقلات والإقامة والمكملات الغذائية، عبئاً إضافياً جوهرياً عليهم. نهدف في هذه المقالة العلمية إلى تقييم النفقات غير الطبية الناجمة عن طلب العلاج من السل، وذلك باستخدام بيانات دراسة مسحية واسعة النطاق.

الطرائق: جرى اختيار 797 حالة إصابة بداء السل من ثلاث مدن بشكل عشوائي باستخدام تصميم شرائحي عنقودي لجمع العينات الكتلة الطبقية تصميم العينات. وجرى حساب التكاليف الطبية لعلاج المرضى المنومين في المشافي، وعلاجهم في العيادات الخارجية، والتكاليف غير الطبية المتعلقة بعلاج مرضى السل، وذلك عن طريق إجراء مقابلات مع المرضى من قبل أشخاص مدربين على ذلك. جرى حساب متوسط والرقم الوسط للتكاليف غير الطبية لعدة مجموعات فرعية ومقارنتها مع بعضها باستخدام اختبارات **U** لـ كاروسكال-واليس و مان-ويتني. كما تم إجراء تحليل انحداري تحليل الانحدار لتقييم تأثير السمات المختلفة للمرضى على مجمل النفقات غير الطبية.

النتائج: بلغ متوسط النفقات غير الطبية 1429 يوان صيني، والمدى الربيعي (مجموعة الربيعي) 424-793 يوان صيني. بلغ متوسط النفقات غير الطبية المرتبطة بعلاج المرضى المنوّمين في المشفى، والمرضى في العيادات الخارجية، والمكملات الغذائية الإضافية 540، 91، 900 يوان صيني على التوالي. ومن بين 797 حالة، جرى التبليغ عن نفقات غير طبية بالغة جداً في20 في المائة من الحالات. كما أظهرت النتائج وجود فوارق جوهرية في الإنفاق بين المدن المختلفة، والفئات العمرية، والمواقع الجغرافية، وبكون الرعاية ضمن المشفى أو في العيادات الخارجية، والمستوى الثقافي والعائلي والمادي للمجموعات البشرية.

الاستنتاجات: تُشكل النفقات غير الطبية المرتبطة بعلاج داء السل عبئاً مادياً ثقيلاً للعديد من مرضى السل. من الضروري تقديم المساعدات المادية الكافية والعاجلة للمرضى، وخاصة الفقراء منهم.

Translated from English version into Arabic by Sari M. Barazi, through

TRANSLATORS
WITHOUT BORDERS

结核病患者的非医疗花费负担情况调查分析

李强，江蔚曦，王全丽，申远，高婧媛，Kaori D. Sato，龙倩，Henry Lucas

摘要

引言：中国结核病患者的诊疗花费一部分由医保承担，一部分由国家项目承担，但患者常常也要承受相当大的自付费用医疗负担。对于结核病患者，特别是贫困结核病患者来讲，非医疗花费（如因诊治结核病带来的交通费、食宿费以及额外的营养费）也是一笔不小的额外负担。本研究的目的是评估结核病患者的非医疗花费经济负担情况。

方法：研究采用横断面调查设计，分层整群随机抽样方法，随机调查了江苏镇江、湖北宜昌、陕西汉中三个市的 797 名结核病患者。通过严格培训过的调查员与患者面对面问卷的调查方式收集到了患者整个诊疗期间的住院、门诊医疗花费，直接支出的非医疗花费。统计分析采用均数中位数描述各组非医疗花费，秩和检验方法进行比较，回归分析方法评价相关影响因素。

结果：患者非医疗花费的中位数是 1429 元，四分位数间距为 424~2793。其中住院治疗、门诊治疗期间的非医疗花费以及治疗期间的营养补充花费中位数分别为 540 元，91 元和 900 元。全部 797 名调查患者中，20%的患者的非医疗花费造成了灾难性支出。不同城市间、年龄组间、城乡间、门诊/住院治疗间、教育水平和家庭收入间患者的非医疗花费均发现统计学显著性差异。
结论：由于结核病诊疗引起的非医疗花费对很多结核病患者造成了严重的经济负担，结核病患者特别是贫困患者急需在治疗期间的给予经济帮助以减轻负担完成治疗。

Translated from English version into Chinese by Li Qiang

Fardeau financier non médical découlant du traitement de la tuberculose : étude transversale en Chine rurale

Qiang Li, Weixi Jiang, Quanli Wang, Yuan Shen, Jingyuan Gao, Kaori D. Sato, Qian Long, Henry Lucas

Résumé

Contexte : En Chine, le traitement de la tuberculose est partiellement couvert par des programmes et des régimes d'assurance-maladie nationaux, mais les malades de la tuberculose doivent souvent faire face à des dépenses de santé considérables. Pour certains, en particulier ceux qui vivent dans des ménages pauvres, les frais non médicaux, comme le transport, le logement et les compléments alimentaires, peuvent constituer un fardeau supplémentaire très important. Cet article a pour objectif d'évaluer ces frais non médicaux découlant du traitement de la tuberculose au moyen de données issues d'une étude transversale à grande échelle.
Méthodes : Au total, 797 cas de tuberculose provenant de trois villes ont été choisis au hasard à l'aide d'un concept d'échantillonnage en grappes stratifié. Les frais médicaux des patients hospitalisés, les frais médicaux des patients en ambulatoire et les frais non médicaux directs découlant du traitement de la tuberculose ont été collectés par le biais d'entretiens directs réalisés par des interrogateurs professionnels. Les frais non médicaux moyens et médians des différents sous-groupes ont été calculés et comparés à l'aide de tests de Kruskal-Wallis et des tests U de Mann-Whitney. Une analyse de la régression a été réalisée pour évaluer l'influence des différentes caractéristiques des patients sur le total des frais non médicaux.
Résultats : Les frais non médicaux médians étaient de 1 429 RMB avec un écart interquartile de 424-2 793 RMB. Les frais non médicaux médians liés au traitement de patients hospitalisés et de patients en ambulatoire, et à l'apport de compléments alimentaires, étaient respectivement de 540, 91, et 900 RMB. Sur les 797 cas, 20 % ont signalé des dépenses de santé catastrophiques pour les frais non médicaux. Des différences statistiques conséquentes ont été détectées entre les différents villes, tranches d'âge, emplacements géographiques, soins en hospitalisation/en ambulatoire, niveaux d'éducation et groupes de revenu familial.
Conclusions : Les frais non médicaux découlant du traitement de la tuberculose constituent un fardeau financier important pour bon nombre de malades de la tuberculose. Il est urgent d'apporter une assistance financière à même de réduire ce fardeau tout au long de la période de traitement, en particulier pour les personnes pauvres.

Translated from English version into French by Claire Michelon, through

TRANSLATORS
WITHOUT BORDERS

Финансовое бремя, не связанное с медицинскими расходами, в лечении туберкулеза: Перекрёстное исследование в сельском Китае

Цян Ли, Вейкси Цзян, Цюаньли Ванг, Юань Шэнь, Цзинюань Гао, Каори Д. Сато, Цянь Лонг, Генри Лукас.

Реферат

История вопроса: Лечение туберкулеза в Китае частично покрывается национальными программами и планами медицинского страхования, но все же больные туберкулезом часто сталкиваются с значительными медицинскими расходами. Для отдельных лиц, особенно происходящих из бедных семей, такие немедицинские расходы, как, например, транспортные расходы, расходы на проживание и дополнительное питание, могут представлять значительное дополнительное бремя. В данной статье мы поставили задачу оценить немедицинские расходы, связанные с лечением туберкулеза, на основе данных, полученных в ходе широкомасштабного перекрёстного исследования.

Методы: Методом районированной гнездовой выборки были произвольно отобраны 797 случаев туберкулеза в трех городах. В ходе личных собеседований, проведенных квалифицированными интервьюерами, были собраны данные по медицинским расходам стационарных и амбулаторных пациентов и по прямым немедицинским расходам, связанным с лечением туберкулеза. Были рассчитаны средние и медианные немедицинские расходы для различных подгрупп, а затем было произведено сравнение с помощью критериев Краскела-Уоллиса и Уилкоксона-Манна-Уитни. Был произведен регрессивный анализ для оценки воздействия различных характеристик пациентов на общие немедицинские расходы.

Результаты: Медианные немедицинские расходы составили 1429 юаней с межквартильным размахом 424 - 2793 юаней. Медианные немедицинские расходы, связанные с лечением стационарных и амбулаторных пациентов и дополнительным питанием, составили RMB 540,91 и 900 юаней соответственно. Из 797 изученных случаев 20% жаловались на непомерные немедицинские расходы. Были обнаружены статистически значимые различия между разными городами, возрастными группами, географическими областями, лечением в стационаре и амбулаторно, уровнями образования и семейного дохода.

Выводы: Немедицинские расходы, связанные с лечением туберкулеза, представляют собой тяжелое финансовое бремя для многих пациентов. Необходима финансовая поддержка на период лечения, призванная сократить такое бремя, особенно для бедных пациентов.

Translated from English version into Russian by Alena Hrybouskaya, through

TRANSLATORS WITHOUT BORDERS

Carga financiera no médica en la atención de la tuberculosis: Una encuesta transversal en la China rural

Qiang Li, Weixi Jiang, Quanli Wang, Yuan Shen, Jingyuan Gao, Kaori D. Sato, Qian Long, Henry Lucas

Resumen

Antecedentes: El tratamiento de la tuberculosis (TB) en China está parcialmente cubierto por los programas nacionales y los planes del seguro de salud, aunque los pacientes con tuberculosis a menudo se enfrentan a considerables gastos médicos. Para algunos, especialmente los de los hogares más pobres, los costos no médicos, como el transporte, el alojamiento y el complemento nutricional pueden ser una considerable carga adicional. En este artículo nos proponemos evaluar estos costos no médicos inducidos por la búsqueda de atención de la TB utilizando datos de una encuesta transversal a gran escala.

Métodos: Se seleccionó al azar un total de 797 casos de tuberculosis de tres ciudades utilizando un diseño de muestreo estratificado y por conglomerados. Se recopilaron los gastos de hospitalización médica, los costos médicos de consultas externas y los costos no médicos directos relacionados con el tratamiento de TB a través de entrevistas personales por entrevistadores entrenados. Se calcularon los costos no médicos promedios y medianos para diferentes subgrupos, utilizando pruebas U de Kruskal-Wallis y Mann-Whitney. Se realizó un análisis de regresión para evaluar la influencia de las diferentes características de los pacientes sobre el total de los costos no médicos.

Resultados: El costo no médico mediano era de 1429 renmibis (RMB), con un rango intercuartil de 424-2,793 renmibis. Los gastos no médicos medianos relacionados con el tratamiento hospitalario, el tratamiento ambulatorio y la nutrición complementaria eran de 540, 91 y 900 renmibis, respectivamente. De los 797 casos, el 20% informó de gastos catastróficos en los costos no médicos. Estadísticamente se detectaron diferencias significativas entre las diferentes ciudades, grupos de edad, ubicaciones geográficas, atención hospitalaria o ambulatoria, niveles de educación y grupos de ingresos familiares.

Conclusiones: Los gastos no médicos relacionados con el tratamiento de la TB son una carga financiera grave para muchos pacientes con tuberculosis. Se necesita con urgencia asistencia financiera que pueda limitar esta carga con urgencia durante el período de tratamiento, especialmente para los pobres.

Translated from English version into Spanish by Susana Rosselli, through

TRANSLATORS
WITHOUT BORDERS
